# Supplementary material for: Acinar to β-like cell conversion through inhibition of focal adhesion kinase
Source: Nat Commun. 2024 May 3;15:3740. doi: 10.1038/s41467-024-47972-4 (PMC11068907; doi:10.1038/s41467-024-47972-4)
Supplement: Supplementary file 1 — Supplementary Information [file 41467_2024_47972_MOESM1_ESM.pdf]

Supplementary Fig. 1

Wild type (FAKi-treated)

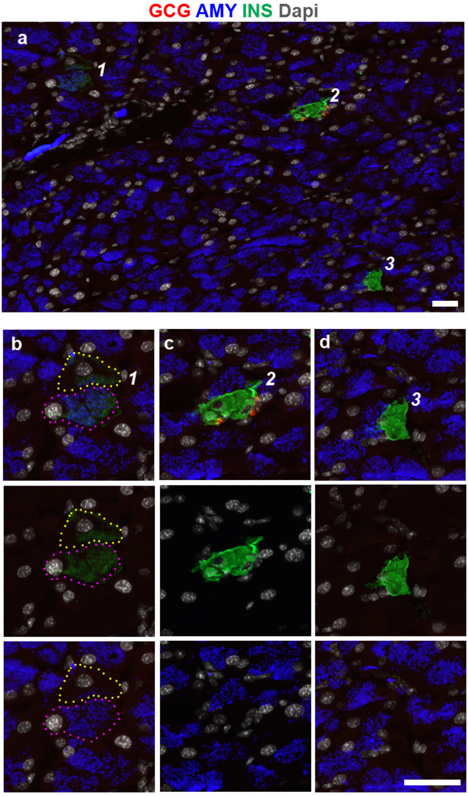

Supplementary Figure 1. Appearance of cells expressing insulin and amylase following FAKi treatment.

(a-d) Representative images of the pancreas obtained from wild type mice treated with FAKi (*n* = 5). Immunostaining for detection of insulin, glucagon and amylase showing clusters of amylase^{+}/insulin^{+} cells **(1 in a and b)**. Yellow and pink dots in **(b)** mark an insulin^{low}/amylase^{-} cell and an adjacent insulin^{low}/amylase^{+} cell, respectively. **(c)** shows a small islet whereas **(d)** highlights two insulin-expressing cells within the acinar compartment. Scale bar = 20 μm.

Supplementary Fig. 2

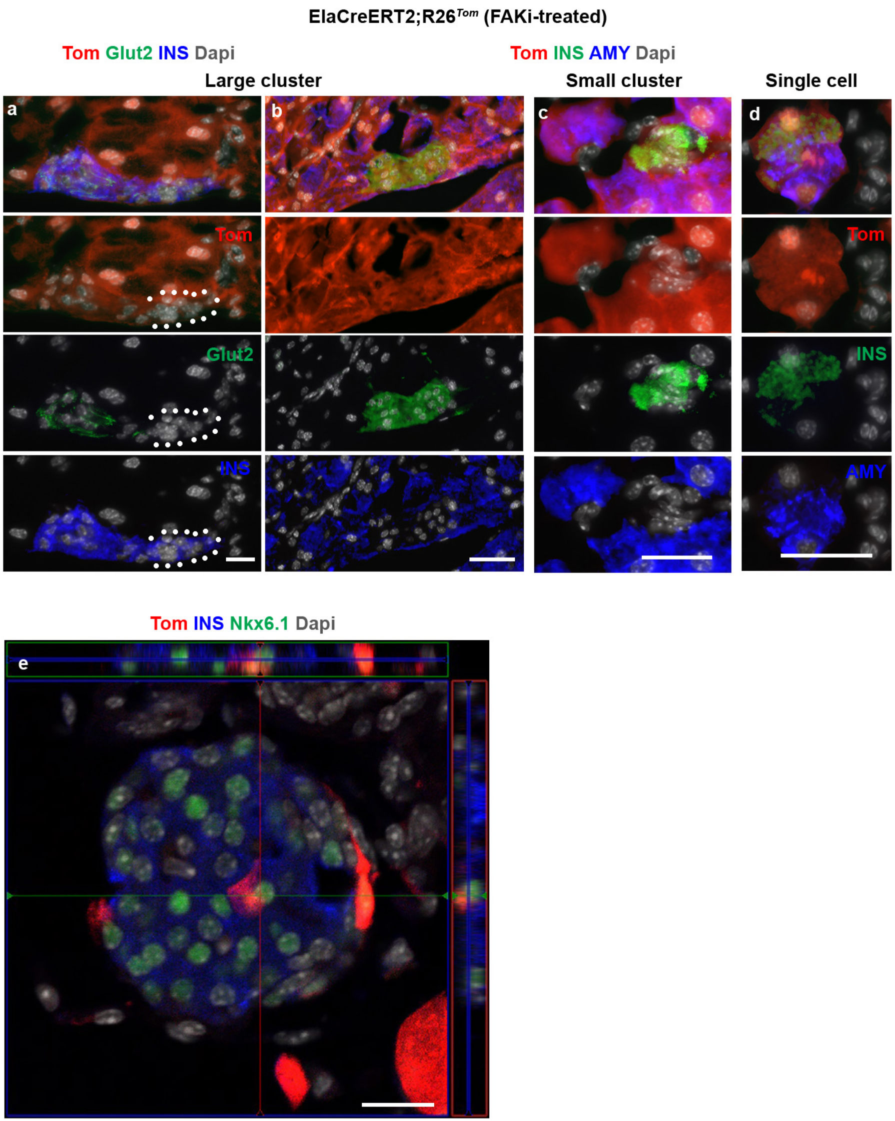

Supplementary Figure 2. Inhibition of FAK leads to acinar lineage conversion to insulin-producing cells.

(a- d) Imaging of tamoxifen-induced ElaCreERT2,R26^{Tom} mice treated with FAKi showing Tom^{+}/insulin^{+} cells outside the endocrine islets. *n* = 5 mice from 3 independent experiments. Fluorescent imaging of Tomato in conjunction with insulin and GLUT2 **(a)**, or insulin and amylase **(b-d)** showing Tom^{+}/insulin^{+} cells in large clusters **(a, b)**, small clusters **(c)**, or single cells **(d)**. Dotted line in **(a)** highlights Tom^{+}/Insulin^{+}/GLUT2^{-} cells next to cells expressing Glut2.

(e) Confocal fluorescent imaging of Tomato in conjunction with insulin and Nkx6.1. Scale bar = 20 µm.

Supplementary Fig. 3

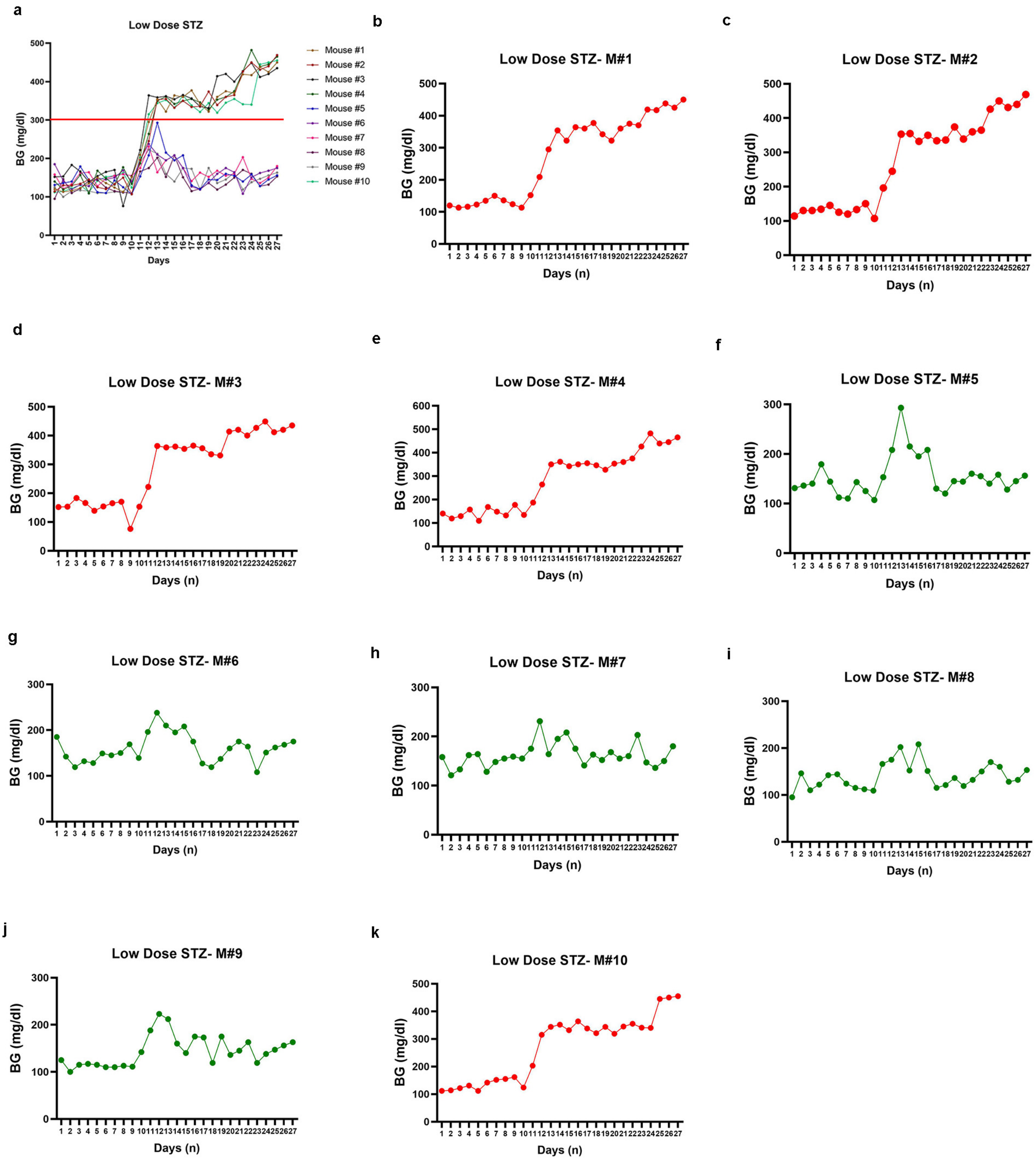

Supplementary Figure 3. Spontaneous blood glucose recovery was observed only in mice with BG below 300mg/dl.

(a-k) Graphs showing combined **(a)** or individual **(b-k)** average weekly BG values based on daily BG measurements in wild type mice treated with low dose STZ (n = 10). Red line in **(a)** mark the critical BG value (300mg/dl) above which no spontaneous recovery could be observed. Source data are provided as a Source Data file.

Supplementary Fig. 4

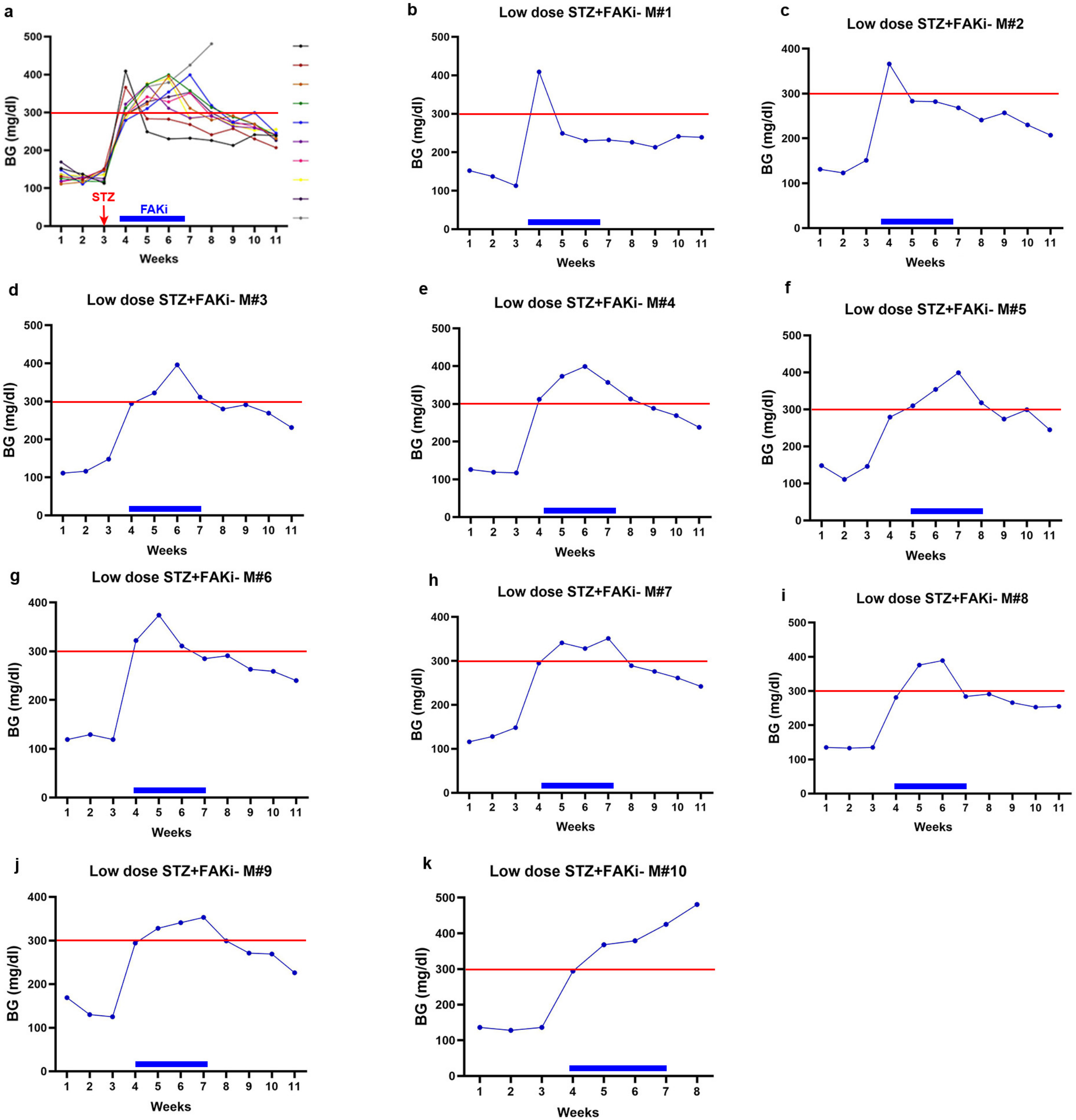

Supplementary Figure 4. FAKi treatment ameliorates blood glucose homeostasis in diabetic mice.

(a-k) Graph showing average weekly BG values based on daily BG measurements in wild type mice treated with low-dose STZ followed by FAKi treatment (*n* = 10 mice).

(b-k) Individual graphs for average weekly BG in wild type mice treated with low-dose STZ followed by FAKi treatment. Red lines mark the critical BG value of 300mg/dl and blue bars on the x-axis denotes the period of FAKi treatment. Source data are provided as a Source Data file.

Supplementary Fig. 5

Wild type mouse (STZ + FAKi)

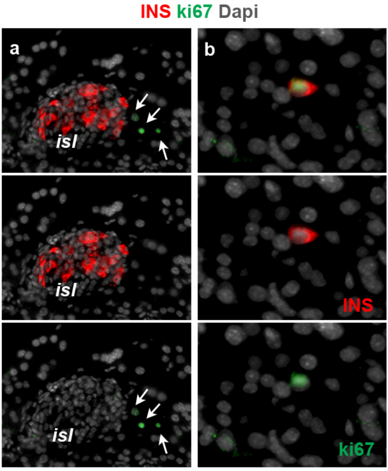

Supplementary Figure 5. Insulin-producing cells within islets do not proliferate.

(a, b) Immunofluorescent imaging for detection of insulin and ki-67 on tissues obtained from wild type mice treated with high-dose STZ followed by FAKi showed single insulin-producing cells that expressed ki-67 (n = 3). Arrows in **(a)** show ki-67^{+} cells next to a cluster of endocrine cells. Scale bar = 20 μm.

Supplementary Fig. 6

a

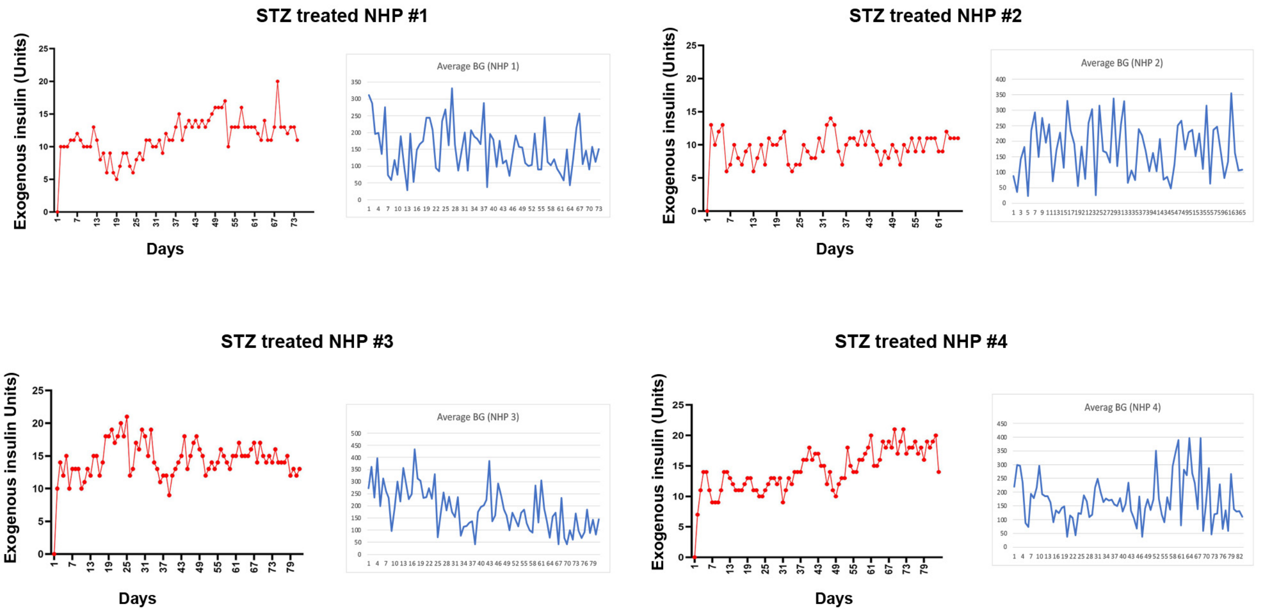

b

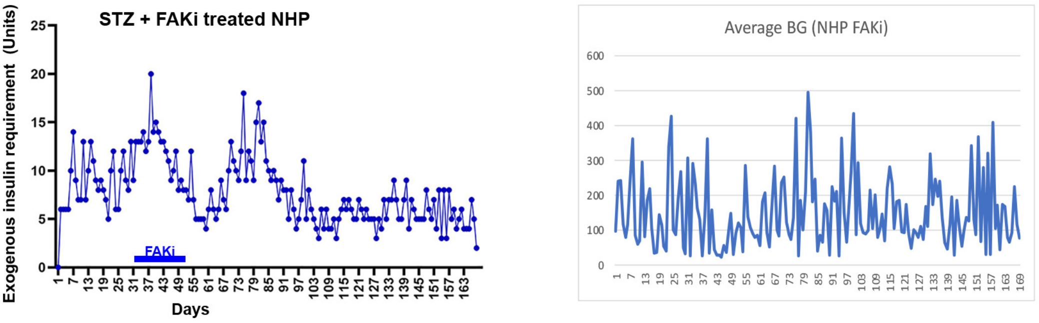

Supplementary Figure 6. FAKi treatment improves blood glucose levels in diabetic NHP.

(a, b) Graphs showing individual average weekly exogenous insulin requirements based on daily measurements in NHPs treated with STZ (n = 4) **(a)** or STZ followed by FAKi treatment (*n* = 1) **(b)**. Data is presented as mean ± SD. Source data are provided as a Source Data file.

Supplementary Fig. 7

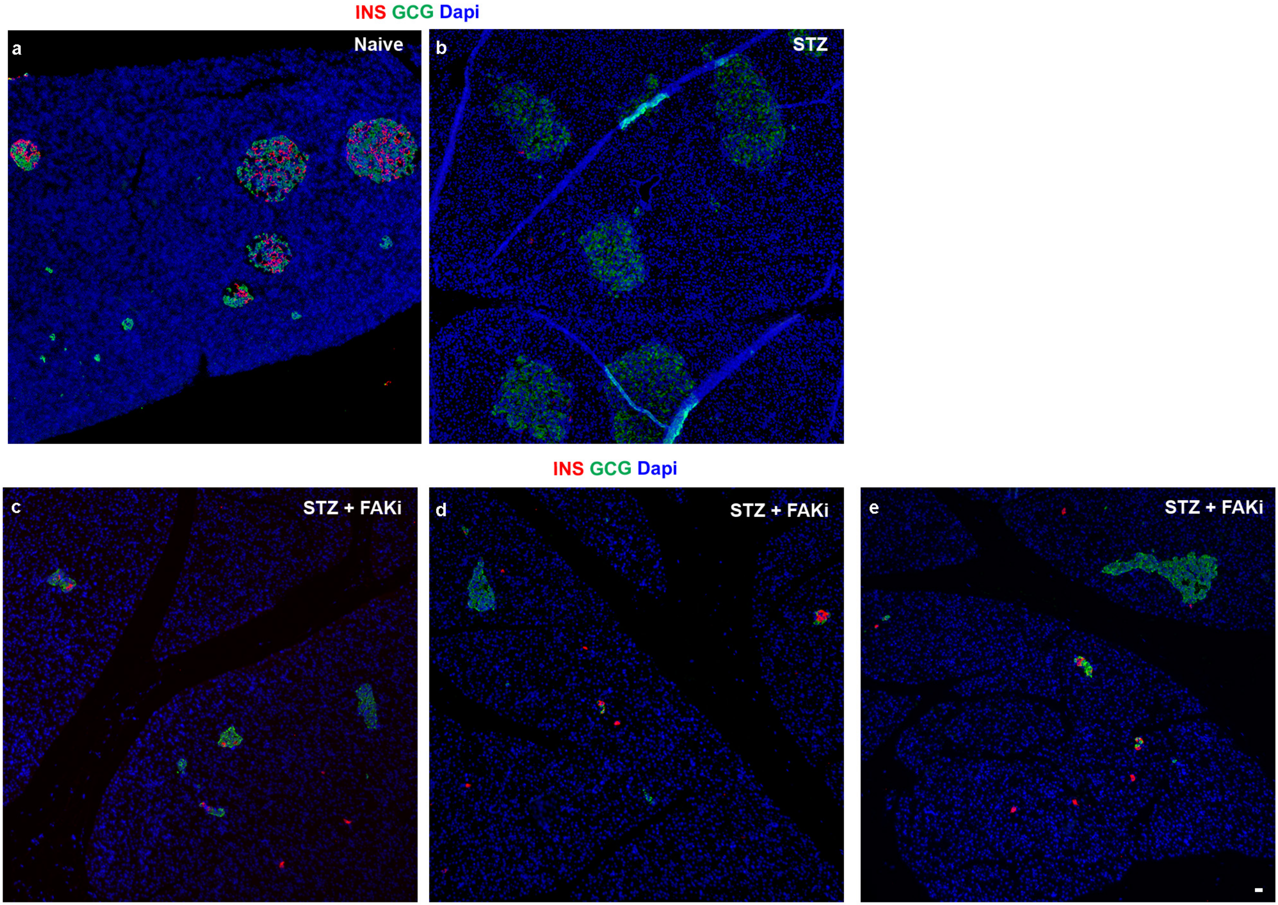

Supplementary Figure 7. FAKi treatment leads to increased β-cell area in NHP following near total β-cell ablation.

(a, b) Low magnification immunofluorescent imaging for detection of insulin and glucagon of representative islets obtained from naïve NHP **(a)**, STZ-treated NHP **(b)** or STZ+FAKi NHP **(c-e)** (*n* = 1 per group).

Supplementary Fig. 8

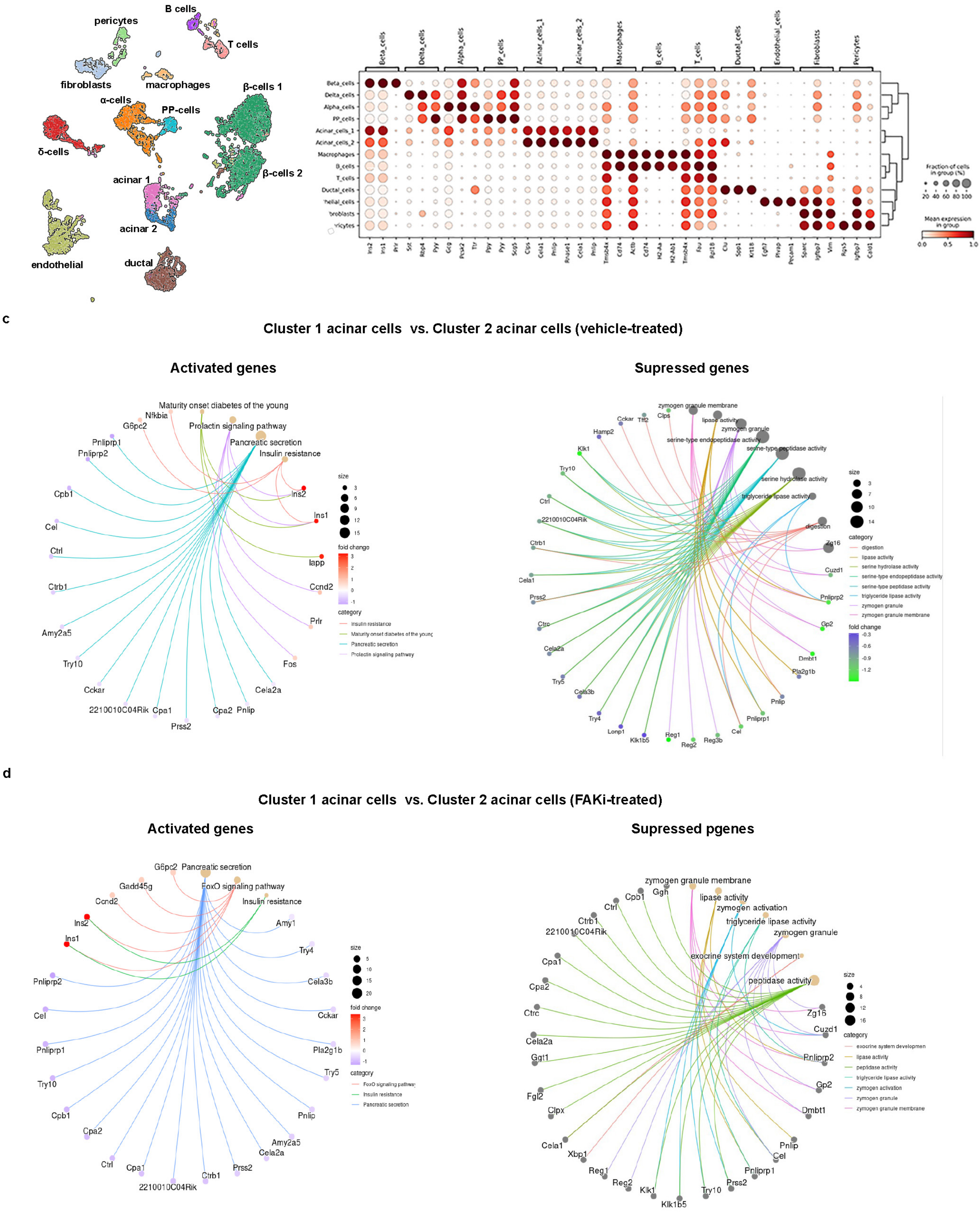

Supplementary Figure 8. scRNA-seq analysis of FAKi-treated acinar- and β-cells.

(a) UMAP of the identified clusters across the single cell dataset defined by specific color.

(**b)** Dot plot showing gene expression of known markers of α-, β-, γ-, PP-, ductal-, acinar-, immune- or endothelial cells.

(c, d) Gene ontology terms of the differentially expressed genes (DEGs) across cluster 1 vs cluster 2 acinar cells in vehicle-treated (**c)** or FAKi-treated (**d)** samples. The cnet plot shows the link between the genes and biological processes, and the color of the genes corresponds to fold change.

Supplementary Fig. 9

a Day 1 post-FAKi treatment

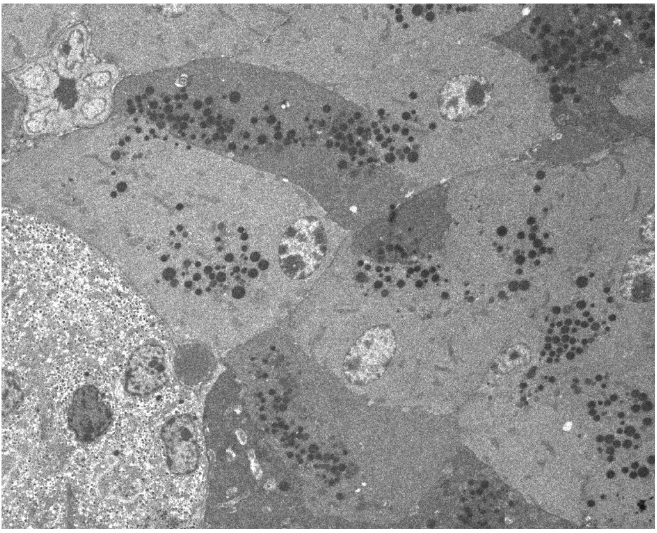

b Day 10 post-FAKi treatment

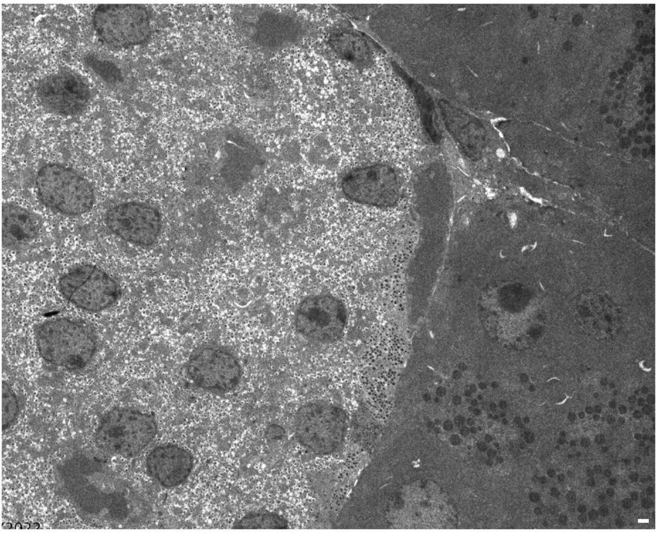

Supplementary Figure 9. Loss of peri-islet hybrid acinar/β-cells following FAKi treatment.

(a, b) TEM of mouse pancreas showing peri-islet area after one **(a)** day and ten days (**b**) of completion of FAKi-treatment. *n* = 3 for each cohort. Scale bar = 2μm**.**
