# Supplementary material for: Acinar to β-like cell conversion through inhibition of focal adhesion kinase
Source: Nat Commun. 2024 May 3;15:3740. doi: 10.1038/s41467-024-47972-4 (PMC11068907; doi:10.1038/s41467-024-47972-4)
Supplement: Supplementary file 3 — Reporting Summary [file 41467_2024_47972_MOESM3_ESM.pdf]

nature portfolio

Corresponding author(s): Farzad Esni
Shakti Dahiya

Last updated by author(s): 2024/03/14

Reporting Summary

Nature Portfolio wishes to improve the reproducibility of the work that we publish. This form provides structure for consistency and transparency in reporting. For further information on Nature Portfolio policies, see our Editorial Policies and the Editorial Policy Checklist.

Statistics

For all statistical analyses, confirm that the following items are present in the figure legend, table legend, main text, or Methods section.

n/a	Confirmed	
☐	☒	The exact sample size (*n*) for each experimental group/condition, given as a discrete number and unit of measurement
☐	☒	A statement on whether measurements were taken from distinct samples or whether the same sample was measured repeatedly
☐	☒	The statistical test(s) used AND whether they are one- or two-sided<br>*Only common tests should be described solely by name; describe more complex techniques in the Methods section.*
☐	☒	A description of all covariates tested
☐	☒	A description of any assumptions or corrections, such as tests of normality and adjustment for multiple comparisons
☐	☒	A full description of the statistical parameters including central tendency (e.g. means) or other basic estimates (e.g. regression coefficient) AND variation (e.g. standard deviation) or associated estimates of uncertainty (e.g. confidence intervals)
☐	☒	For null hypothesis testing, the test statistic (e.g. *F*, *t*, *r*) with confidence intervals, effect sizes, degrees of freedom and *P* value noted<br>*Give P values as exact values whenever suitable.*
☒	☐	For Bayesian analysis, information on the choice of priors and Markov chain Monte Carlo settings
☒	☐	For hierarchical and complex designs, identification of the appropriate level for tests and full reporting of outcomes
☒	☐	Estimates of effect sizes (e.g. Cohen's *d*, Pearson's *r*), indicating how they were calculated

Our web collection on statistics for biologists contains articles on many of the points above.

Software and code

Policy information about availability of computer code

Data collection	CellRanger (V6.0172)<br>Seurat (V3.1, R package)<br>LASX software (1.4.5 277713)<br>Adobe Photoshop (25.3.1)<br>Philips EM 208
Data analysis	Image J<br>Graph Pad Prism software (version 9.2.0)<br>Microsoft Excel (16.77.1)

For manuscripts utilizing custom algorithms or software that are central to the research but not yet described in published literature, software must be made available to editors and reviewers. We strongly encourage code deposition in a community repository (e.g. GitHub). See the Nature Portfolio guidelines for submitting code & software for further information.

Data

Policy information about availability of data

All manuscripts must include a data availability statement. This statement should provide the following information, where applicable:

- Accession codes, unique identifiers, or web links for publicly available datasets
- A description of any restrictions on data availability
- For clinical datasets or third party data, please ensure that the statement adheres to our policy

The RNAseq data generated in this study have been uploaded to the Gene Expression Omnibus under accession number: GSE251852 (https://www.ncbi.nlm.nih.gov/geo/query/acc.cgi?&acc=GSE251852).
Source Data files underlying the graphs are presented in Supplementary information.

Research involving human participants, their data, or biological material

Policy information about studies with human participants or human data. See also policy information about sex, gender (identity/presentation), and sexual orientation and race, ethnicity and racism.

Reporting on sex and gender	N/A
Reporting on race, ethnicity, or other socially relevant groupings	N/A
Population characteristics	N/A
Recruitment	N/A
Ethics oversight	N/A

Note that full information on the approval of the study protocol must also be provided in the manuscript.

Field-specific reporting

Please select the one below that is the best fit for your research. If you are not sure, read the appropriate sections before making your selection.

☒ Life sciences ☐ Behavioural & social sciences ☐ Ecological, evolutionary & environmental sciences

For a reference copy of the document with all sections, see nature.com/documents/nr-reporting-summary-flat.pdf

Life sciences study design

All studies must disclose on these points even when the disclosure is negative.

Sample size	Sample sizes were chosen based on previously published literature of the similar subject (PMID:34582892, PMID:33351784 and PMID:32122971) as well as the evaluation of animal ethics versus ability to obtain sufficient samples for statistical analysis. AUC for GTT was calculated by the trapezoidal method. Comparisons between 2 groups were made using unpaired, 2-tailed t-test as indicated. Comparison between multiple groups was made using one way AN OVA followed by the Holm-Sid.k test for multiple comparisons. Significance was presented as asterisks, calculated by Graph Pad Software, as described under Methods.
Data exclusions	No data were excluded.
Replication	All attempts at replication of data included in this manuscript were successful. Biological replicates were at least n=5 unless otherwise stated. These are clearly shown with dot-plot overlaying the bar graphs.
Randomization	There was no randomization as there were no clinical studies nor patient participants were involved. Aged matched animals were randomly allocated to groups for different treatments (saline, STZ, vehicle or FAKi).
Blinding	For the lineage tracing studies, the investigators were not blinded to allocations during experiments or analysis, as Tomato+ cells invading islets (with or without insulin expression) as the result of FAKi treatment were clearly visible and totally absent in the vehicle-treated cohort. For the treatment of diabetic mice, the researchers who treated the mice with saline, STZ, FAKi, or vehicle knew the type of treatment and concentration used. However, the researchers who performed the blood glucose analysis or GTT were blinded to the allocated groups. For the NHP studies, FAKi treatment, daily blood glucose measurements and daily insulin dose determination and administration were all conducted by technical staff. For the EM studies, the researchers analyzing the images were blinded to the allocated groups. The investigator performing the bioinformatic analysis was initially blinded to the allocated groups.

Reporting for specific materials, systems and methods

We require information from authors about some types of materials, experimental systems and methods used in many studies. Here, indicate whether each material, system or method listed is relevant to your study. If you are not sure if a list item applies to your research, read the appropriate section before selecting a response.

Materials & experimental systems

n/a	Involved in the study
☐	☒ Antibodies
☒	☐ Eukaryotic cell lines
☒	☐ Palaeontology and archaeology
☐	☒ Animals and other organisms
☒	☐ Clinical data
☒	☐ Dual use research of concern
☒	☐ Plants

Methods

n/a	Involved in the study
☒	☐ ChIP-seq
☒	☐ Flow cytometry
☒	☐ MRI-based neuroimaging

Antibodies

Antibodies used	goat anti-Amylase (1:2S0, Santa Cruz, sc-12821)<br>rat anti BrdU (1:100, Abeam, ab6326)<br>rabbit anti-Glucagon (1:1000, Linco/Millipore, 4031-0lF)<br>rabbit anti-Glut2 (1:50, Santa Cruz, sc-31825)<br>guinea pig anti-Insulin (1:400, Abeam, ab195956)<br>rabbit anti-Insulin (1:500, Abeam, ab181547)<br>rat anti ki-67 (1:250, lnvitrogen, 14-5698-82)<br>rabbit anti-Nkx6.l (1:150, Abeam, ab221549)<br>goat anti-Pdxl (1:100, Abeam, ab47383)<br><br>The following secondary antibodies were purchased from Jackson lmmunoResearch Laboratories:<br>biotin-conjugated anti-rabbit (1:500, 711-066-152), biotin-conjugated anti-rat (1:500, 712-066-153), biotin conjugated anti-guinea pig (1:500, 706-065-148), biotinconjugated anti-goat (1:250, 705-065-147); Cy2-conjugated streptavidin (1:500, 016-540- 084); Cy3-conjugated streptavidin (1:500, 016-160-084); Cy5-conjugated streptavidin (1:100, 016-600-084); Cy2-conjugated anti-guinea pig (1:300, 706-545-148), Cy3-conjugated anti-guinea pig (1:300, 706-166-148), Cy2-conjugated anti-rabbit (1:300, 711-485-152), Cy3-conjugated anti-rabbit (1:300, 711-165-152), Cy2-conjugated anti-rat (1:300, 712-545-153), Cy3-conjugated anti-rat (1:300, 712-166-150), Cy2-conjugated anti-goat (1:300, 705-545-147) and Cy3-conjugated anti-goat (1:300, 705-165 147).
Validation	All antibodies used have been purchased from vendors and have been validated in previous reports.<br>Amylase (PMID:2425877)<br>BrdU (PMID:36961817)<br>Glucagon (PMID:33906911)<br>Glut2 (PMID:29587416)<br>Guinea pig Insulin (PMID:21763240)<br>Rabbit insulin (PMID:36017799)<br>Ki-67 (PMID:26949251)<br>Nkx6.1 (PMID:33723463)<br>Pdx1 (PMID:35798741)

Animals and other research organisms

Policy information about studies involving animals; ARRIVE guidelines recommended for reporting animal research, and Sex and Gender in Research

Laboratory animals	Species: Mus musculus and non-human primates.<br>Mouse strains: 2-6 months old C57BL/6, CDl and other genetiacally modified animals on a mixed background: ElaCreERT2, Fak-floxed, Rosa26-CAGTomato.<br>NHP: 4-6 years old male and female Cynomolgus macaques.
Wild animals	This study did not involve wild animals.
Reporting on sex	Equal number of female and male mice were used, except for the studies involving diabetic mice, where age-matched CDl female mice were used. To avoid the differences between sexes, the in vivo glucose homeostasis studies require comparison studies on age and sex-matched groups. Thus, we chose only females.
Field-collected samples	This study did not involve samples collected from fields.
Ethics oversight	Mice and non-human primates (NHPs) used in these studies were maintained according to protocols approved by the University of Pittsburgh Institutional Animal Care and Use Committee.

Note that full information on the approval of the study protocol must also be provided in the manuscript.

Plants

Seed stocks	N/A
Novel plant genotypes	N/A
Authentication	N/A
